# Pre-Work for the Birth of Driver-Less Scraper (LHD) in the Underground Mine: The Path Tracking Control Based on an LQR Controller and Algorithms Comparison

**DOI:** 10.3390/s21237839

**Published:** 2021-11-25

**Authors:** Haoxuan Yu, Chenxi Zhao, Shuai Li, Zijian Wang, Yulin Zhang

**Affiliations:** School of Resources and Safety Engineering, Central South University, Changsha 410083, China; yuhaoxuan@csu.edu.cn (H.Y.); 8210183019@csu.edu.cn (C.Z.); 18973155503@163.com (Z.W.); 8210183024@csu.edu.cn (Y.Z.)

**Keywords:** scraper (LHD), artificial intelligence, path tracking, mining engineering

## Abstract

With the depletion of surface resources, mining will develop toward the deep surface in the future, the objective conditions such as the mining environment will be more complex and dangerous than now, and the requirements for personnel and equipment will be higher and higher. The efficient mining of deep space is inseparable from movable and flexible production and transportation equipment such as scrapers. In the new era, intelligence is leading to the development trend of scraper (LHD), path tracking control is the key to the intelligent scraper (LHD), and it is also an urgent problem to be solved for unmanned driving. This paper describes the realization of the automatic operation of articulating the scraper (LHD) from two aspects, a mathematical model and trajectory tracking control method, and it focuses on the research of the path tracking control scheme in the field of unmanned driving, that is, an LQR controller. On this basis, combined with different intelligent clustering algorithms, the parameters of the LQR controller are optimized to find the optimal solution of the LQR controller. Then, the path tracking control of an intelligent LHD unmanned driving technology is studied, focusing on the optimization of linear quadratic optimal control (LQR) and the intelligent cluster algorithms AGA, QPSO, and ACA; this research has great significance for the development of the intelligent scraper (LHD). As mining engineers, we not only need to conduct research for practical engineering projects but also need to produce theoretical designs for advanced mining technology; therefore, the area of intelligent mining is the one we need to explore at present and in the future. Finally, this paper serves as a guide to starting a conversation, and it has implications for the development and the future of underground transportation.

## 1. Introduction

### 1.1. Retrospective: Development of Underground Driver-Less Technology

With the gradual rise of driver-less technology and artificial intelligence, driver-less electric vehicles have been significantly applied in all walks of life.

The earliest driver-less technology was envisioned for life driving: one of the earliest cases of driver-less technology was the CMU (Carnegie Mellon University) system [1,2], in which a system based on artificial intelligence computing and the computer control was developed by CMU (Carnegie Mellon University). Subsequently, quite a few scholars have developed more and more control systems based on the CMU system, providing favorable technical support for the realization of unmanned driving [3,4]. After the 2010s, Google [5,6], Uber [7], Baidu [8], Huawei [9], and other giant enterprises have all participated in the development of self-driving cars, which undoubtedly drives the development of self-driving technology. At the same time, the impact of driver-less technology on industrial transportation is also significant, and mining transportation is no exception. In mining operations, especially in underground mining and transportation operations, it is difficult to optimize the route planning and scheduling of transport vehicles, but in recent years, the introduction of unmanned driving technology had made mining and transportation full of new “spring”.

The track-less Applied Automatic Guidance vehicles (AGVs) are the most widely used driver-less vehicles in the field of industrial transportation. In 2011, Bellamy and Pravica [10] explored the importance of introducing driver-less technology to the Australian open-pit mining industry for transportation; here, unmanned (driver-less) rail transportation technology in underground mines is the earliest and fastest developing direction, and many researchers have made great achievements in this direction. Around the year 2020, H. Yu, S. Li, and J. Yu [11,12] first proposed the application of the Communication-Based Train Control (CBTC) system in the intelligent monitoring system of underground mine rail transportation. Their proposals and ideas have been widely recognized, so more and more researchers who are doing research on mining transportation began to explore the application of CBTC systems in underground mines [13,14,15].

**Figure 1:**
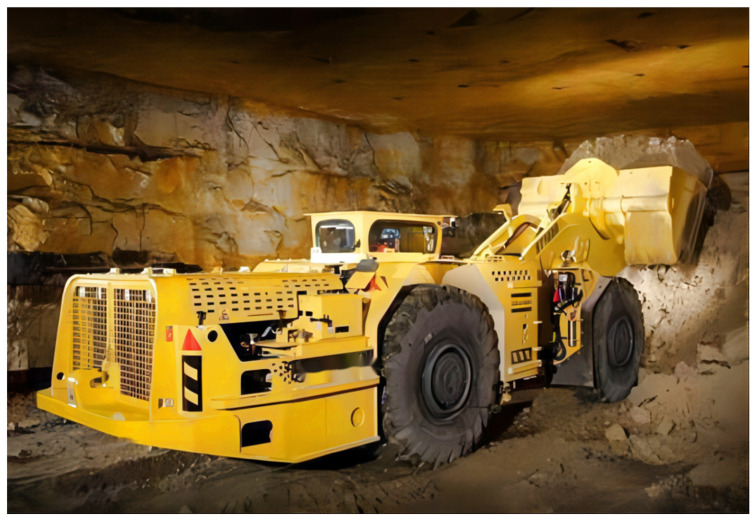
Track-less transport vehicle: the underground scraper (LHD).

Rail transportation is convenient, but there are many inconveniences for loading and unloading equipment, especially for the underground scraper, which is also called load–haul–dump (LHD). For loading and unloading equipment, flexibility is the most important matter taken into account, as shown in Figure 1.

### 1.2. Retrospective: Development of Driver-Less Scraper (LHD) in the Underground Mine

With the development of industry, the global demand for mineral resources continues to soar. At the same time, when surface resources are increasingly exhausted, the mines will develop in the direction of deep sea, deep ground, and deep space in the future. As a kind of large trackless equipment, the scraper plays an important role in the production of underground metal mines and directly reflects the technical level and production capacity of modern underground mining. For example, the scraper can replace manual work in the deep underground space and other harsh environments, which greatly improves the efficiency of mining.

In recent years, due to the development of excavating equipment and the construction of intelligent mines, the development of scrapers is rapid; most mines have begun to apply more advanced remote control scrapers and have achieved favorable practical results [16,17]. A scraper with remote control enables not only simple manipulation, it is also safe, and the advent of the era of 5G, the rapid development of artificial intelligence technology, and the application of robot control technique have made scrapers again an innovation. enabling further development on the basis of the remote control scraper humanization service sector. Soon, scrapers will include artificial intelligence, so as to realize self-driving technology. As one of the core problems in the field of unmanned intelligent LHD, the path tracking control technology of the LHD is proposed.

In 2017, Y. Jiang et al. [18] proposed a particle swarm optimization-based underground intelligent scraper (LHD) motion trajectory control method. They established a simulation model of the entire system in Simulink according to the autonomous navigation dual-variable PID control algorithm and the scraper motion control model. Subsequently, in 2018, J. Li and K. Zhan [19] analyzed the research status and development trend of intelligent technology in underground metal mines in China, and they especially systematically reviewed the development status and trend of intelligent scrapers (LHD). As early as 2008, C. Yin et al. [20] proposed an intelligent optimization method of LHD path tracking for underground mining; in the same year, 2008, T. Hu et al. [21] proposed an intelligent shovel control strategy for LHD used in underground mining. These are early studies on intelligent LHD.

Recently, Q. Gu et al. [22] proposed an autonomous scraper (LHD) trajectory planning method based on numerical optimization according to the most common transport and loading scenes of underground mines, providing a safe and feasible space–time trajectory for efficient production. Y. Meng et al. [23] also studied the path tracking control of an intelligent scraper (LHD), and their research also provided some enlightenment to us. In 2021, it was reported that [24] Sandvik developed intelligent scrapers (LHD) with a color screen that should be in production within the next five years. Additionally, a control strategy of the LHD is proposed to replace the human control with the computer control; that is, the LHD can accurately operate according to the instruction when receiving the instruction. This not only ensures the normal operation of underground production and transportation operation, but also the intelligent underground transportation system will greatly improve the production capacity of mine [25].

Nevertheless, in order to further promote the development of LHD, the research of path following control still needs a certain theoretical and practical basis [26]. In addition, to make the intelligent scraper more widely used in the future, problems such as the influence of high depth, high temperature, and high stress on the operation of the scraper in deep mining require being solved urgently.

Now, green mining has become the theme of mining in the new era, and the development of green mining is inseparable from the research of intelligent mining, including the continuous innovation of scrapers [27]. On the whole, the development of the intelligent scraper is the indispensable result of the development of mining equipment. At the same time, continuous innovation and development of intelligent scrapers will also make grand contributions to the construction of green mines.

Therefore, based on previous research [18,19,20,21,22,23], we, mining researchers on the front lines, focusing on the path tracking control of the driver-less technology of the intelligent scraper (LHD), mainly studied the optimization of linear quadratic optimal control (LQR) and intelligent cluster algorithms AGA, QPSO, and ACA. This research has great significance for the development of the intelligent scraper (LHD).

## 2. Materials and Methods

### 2.1. Mathematical Model of Underground Articulated LHD (Scraper)

In terms of mathematical modeling, the kinematics model of the underground articulated scraper has been widely used in the field of path tracking due to its simple motion mechanism and the ability to obtain an accurate model [28].

#### 2.1.1. Kinematics Model of Articulated Scraper

The underground intelligent scraper belongs to the articulated car [29]. The car body is divided into front and rear ends, the front end and the rear end are connected by the hinge point [30]. In the deep tunnel environment, the car body has a small steering radius; thus, it is more flexible.

**Figure 2:**
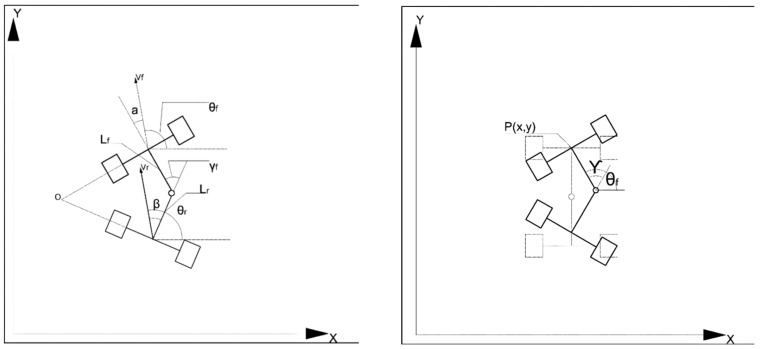
The rotating model of an articulated scraper.

To determine the geometric relationship between the real-time position change information and the motion variables of the LHD, it is essential to analyze the kinematics of the underground articulated LHD and establish the kinematics equation [31]. The body structure of the underground articulated scraper is shown in Figure 2 above and below.

In the coordinate system, the front part of the car body is *P*_1_(*X*_1_,*Y*_1_), the rear part of the car body is *P*_2_(*X*_2_,*Y*_2_); the center of mass velocity of the front body is v_1_, and the center of mass velocity of the rear body is v_2_; the length of the front body is L_1_, and the length of the rear body is L_2_; the slip angle of the front body is $\alpha_{1}$, and the slip angle of the rear body is $\alpha_{2}$; the heading angle of the front body is ${\dot{\theta }}_{1}$ and the heading angle of the rear body is ${\dot{\theta }}_{2}$; the heading angular velocity of the front body is expressed as ${\dot{\theta }}_{1}$, and the heading angular velocity of the rear body is expressed as ${\dot{\theta }}_{2}$.

Define the course angular velocity:

(1) $$\dot{\gamma }=\dot{\theta_{1}}-{\dot{\theta }}_{2}.$$

Under normal circumstances, the operation speed of the underground scraper is slow and generally will not be over 30 km/h [32]. Provided that the influence of tire deformation and vehicle body slip is ignored, that is, $\alpha_{1}$ = $\alpha_{2}$, then the motion state model of the underground articulated scraper is [33]:

(2) $$\{\begin{array}{c} -{\dot{x}}_{1}sin\theta_{1}+{\dot{y}}_{1}cos\theta_{1}=0\\ -{\dot{x}}_{2}\sin(\theta_{2}+\gamma )+{\dot{y}}_{2}\cos\left(\theta_{2}+\gamma \right)\\ {\dot{\theta }}_{2}\left(L_{1}+L_{2}cos\gamma \right)+\dot{\gamma }L_{1}=0\\ -v_{1}+v_{2}cos\gamma +{\dot{\theta }}_{2}L_{2}sin\gamma =0 \end{array}.$$

Note: $\dot{\gamma }$ is the articulated angular velocity.

By selecting the midpoint of the front axle as the reference point of the vehicle state, the kinematics model of the underground articulated scraper can be obtained as:

(3) $$\left[\begin{array}{c} \dot{x}\\ \dot{y}\\ {\dot{\theta }}_{1}\\ \dot{\gamma } \end{array}\right]=[\begin{array}{c} cos\theta_{1}\\ sin\theta_{1}\\ \frac{sin\theta_{1}}{L_{1}cos\gamma +L_{1}}\\ 0 \end{array}\begin{array}{c} 0\\ 0\\ \frac{\begin{array}{c} L_{1} \end{array}}{L_{1}cos\gamma +L_{1}}\\ 1 \end{array}]\left[\begin{array}{c} v_{1}\\ \dot{\gamma } \end{array}\right].$$

According to Equation (3), the motion state of the whole vehicle body can be controlled by the articulated angular velocity of the front car body of the underground articulated scraper [34].

#### 2.1.2. Location Prediction Model 

Using prediction location of the current state of movement to solve the motion state of the next moment, the control strategy introduced in predicting the location can be appropriate to control the amount of compensation in advance, reduce the error of the controller in the future and thus enable more reasonable control output, avoid overcontrol and excessive control, and ensure the quality of underground articulated scarper (LHD) path tracking control [35].

**Figure 3:**
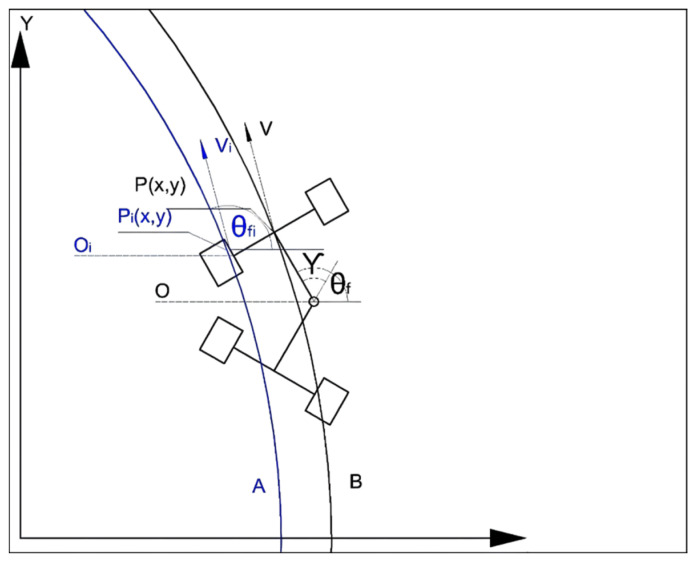
Motion track diagram of the underground articulated scraper.

An articulated vehicle running curve and the parameter definition are shown in Figure 3. Curve A is the ideal path of the underground articulated scraper.

Regarding the ideal path of track curve A and the actual path of track curve B, $v_{i}$ is the ideal speed of the articulated scraper, and v is the actual running speed of the articulated scraper; $\theta_{i}$ is the ideal heading angle of articulated scraper, and θ is the actual heading angle of articulated scraper; $P_{i}\left(x,y\right)$ is the ideal reference point of the articulated scraper, and P(x,y) is the actual reference point of the articulated scraper; $O_{i}$ is the ideal steering center of the articulated scraper, and O is the actual steering center of the articulated scraper [36].

In addition, the center of the front car body of the underground articulated scraper is *P*, and the predicted position point of the scraper is $P_{i}$; the steering center of the front car body is $\theta_{1}$, and the steering center of the rear car body is $\theta_{2}$; the steering radius of the front body is $R_{1}$, and the steering radius of the rear body is $R_{2}$.

According to the motion equation of the articulated vehicle, the change rate of the front steering angle of the vehicle is:

(4) $${\dot{\theta }}_{2}=\frac{v_{1}sin\gamma +L_{2}\dot{\gamma }}{L_{1}cos\gamma +L_{2}}.$$

Set the sampling interval as △t, and the predicted heading angle of the front end of the vehicle is:

(5) $$\theta_{1}{}^{\prime }=\theta_{1}+\frac{v_{1}sin\gamma +L_{2}\dot{\gamma }}{L_{1}cos\gamma +L_{2}}△t.$$

Assuming that the clockwise rotation direction of the vehicle body is the opposite direction, the solution can be obtained:

(6) $$R_{1}=\frac{v_{1}\left(L_{1}cos\gamma +L_{2}\right)}{v_{1}sin\gamma +L_{2}\dot{\gamma }}.$$

To distinguish the rotating motion state of the front car body from the linear motion state of the rear car body, the steering state is set as $t_{0}$, and the following conditions are satisfied:

(7) $$t_{0}=\{\begin{array}{c} 1\;(r>\varepsilon )\\ -1\;(r<-\varepsilon )\\ 0\;(-\varepsilon <r<\varepsilon ) \end{array}$$

where the threshold value is selected to be small. When the degree of steering angle γ of the underground articulated LHD falls within the range of the threshold value, it can be assumed that the LHD does not rotate [37]. When the underground articulated scraper moves from P(x,y) to $P^{\prime }(x^{\prime },y^{\prime })$, the time taken is △t, and the change rate of the steering angle of the front segment of the vehicle is $w_{1}$; then, the angle of the front segment of the vehicle turn is:

(8) $$\gamma^{\prime }=\left|w_{1}\right|△t.$$

According to the geometric relation, the forward distance of the vehicle can be calculated:

(9) $$l=2R_{1}sin\frac{\gamma^{\prime }}{2}.$$

It can be deduced that the midpoint $P^{\prime }(x^{\prime },y^{\prime })$ of the front end of the car body in the next period is:

(10) $$\delta =\{\begin{array}{c} 0\;\left(\theta_{f}=\theta_{f}\right)\\ \theta_{1}+t_{0}\left(\gamma^{\prime }/2\right)\;(0<\theta_{f}<\pi /2)\\ \pi -\theta_{1}-t_{0}\left(\gamma^{\prime }/2\right)\;\left(\pi /2\leq \theta_{f}\leq \pi \right)\\ -\theta_{1}-t_{0}\left(\gamma^{\prime }/2\right)\;\left(-\pi /2\leq \theta_{f}\leq 0\right)\\ \pi +\theta_{1}+t_{0}\left(\gamma^{\prime }/2\right)\;\left(-\pi \leq \theta_{f}\leq -\pi /2\right) \end{array}$$

(11) $$y^{\prime }=\{\begin{array}{c} y+v_{1}△tsin\theta_{1}\\ y+lsin\delta \;(0<\theta_{f}<\pi /2)\\ y+lsin\delta \;\left(\pi /2\leq \theta_{f}\leq \pi \right)\\ y-lsin\delta \;\left(-\pi /2\leq \theta_{f}\leq 0\right)\\ y-lsin\delta \;\left(-\pi \leq \theta_{f}\leq -\pi /2\right) \end{array}$$

(12) $$x^{\prime }=\{\begin{array}{c} x+v_{1}△tcos\theta_{1}\\ x+lcos\delta \;(0<\theta_{f}<\pi /2)\\ x-lcos\delta \;\left(\pi /2\leq \theta_{f}\leq \pi \right)\\ x+lcos\delta \left(-\pi /2\leq \theta_{f}\leq 0\right)\\ x-lcos\delta \left(-\pi \leq \theta_{f}\leq -\pi /2\right) \end{array}.$$

Note: δ is the deviation between the predicted heading angle and the heading angle of the current position.

#### 2.1.3. Deviation Dynamics Model

**Figure 4:**
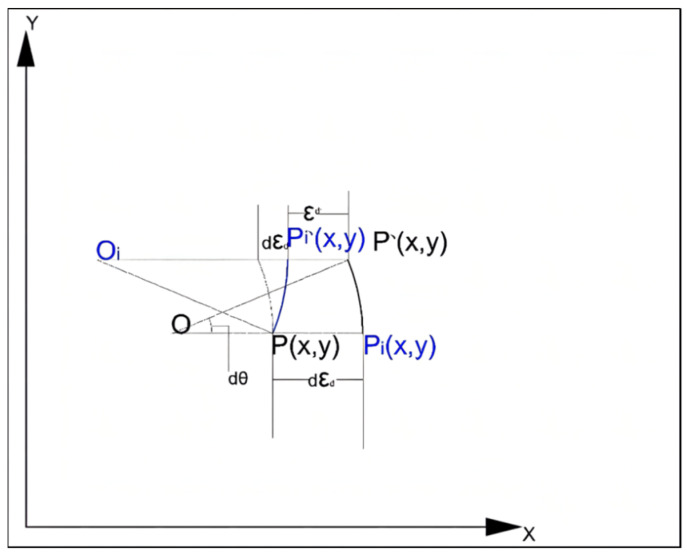
Error dynamic model of the articulated scraper.

The core of Part I is to take the speed and hinge angle of the underground articulated scraper in the current control scheme as the main control variables, so as to realize the control of the state of the hinge angle [38]. Based on the predicted heading angle and the current predicted heading deviation, the control system can effectively improve the control accuracy of reaction path tracking [39]. The average moving speed of the vehicle body is *v*, and the kinematic equation can be calculated according to the error obtained by comparing the actual path of the underground articulated scraper with the ideal path. The error model of the underground articulated scraper is shown in Figure 4.

Position deviation $\delta_{1}$ is the lateral position error between the reference point *P* of the underground articulated scraper and the relative point *P* on the planned path:

(13) $$\dot{\delta_{\begin{array}{c} 1 \end{array}}}=v\delta_{\begin{array}{c} 2 \end{array}}.$$

Driving direction deviation $\delta_{0}$ is the difference between the direction angle of the reference location point *P* of the underground articulated scraper and the direction angle of the reference location point *P* on the ideal motion trajectory:

(14) $$\dot{\delta_{2}}=v\delta_{\begin{array}{c} 3 \end{array}}+\dot{r}\frac{L_{2}}{L_{2}+L_{1}cosr}.$$

Curvature deviation is the curvature error between the reference locus *P* of the underground articulated scraper and the reference locus *P* of the locus:

(15) $$\dot{\delta_{3}}=\frac{v\left(L_{1}+L_{r}cosr\right)\dot{r}+L_{2}\left(L_{2}+L_{1}cosr\right)\ddot{r}+\left(L_{1}L_{2}sinr\right){\dot{r}}^{2}}{v{\left(L2+L_{1}cos\gamma \right)}^{2}}.$$

According to Equations (13)–(15) and *L = L*_1_ + *L*_2_, it can be obtained:

(16) $$\left\{\begin{array}{c} \dot{\varepsilon_{1}}\\ {\dot{\varepsilon }}_{2}\\ \dot{\varepsilon_{3}} \end{array}\right\}=\left[\begin{array}{ccc} 0 & v & 0\\ 0 & 0 & v\\ 0 & 0 & 0 \end{array}\right]\left\{\begin{array}{c} \varepsilon_{1}\\ \varepsilon_{2}\\ \varepsilon_{3} \end{array}\right\}+\left\{\begin{array}{c} 0\\ 0\\ \left(\frac{1}{L}\right) \end{array}\right\}\;\dot{\gamma }+\left\{\begin{array}{c} 0\\ 0\\ \left(\frac{L_{1}}{vL}\right) \end{array}\right\}\ddot{\gamma }.$$

Since the underground roadway is narrow, the underground scraper can only run at a low speed when the two sides of the roadway are close to each other [40]. Therefore, the real-time control of the hinged steering angle of the underground scraper is the key and difficult point during the path tracking. To realize the path tracking of the LHD in the roadway, the steering angular velocity and speed of the articulated angle were selected as the control variables, and the error dynamics model was established based on the actual path and expected path of the LHD [41]. The speed of the articulated vehicles is slow, the change of the articulated angle is small, and the articulated angular acceleration is generally negligible. Therefore, the deviation equation in the above equation is simplified, and the deviation dynamic equation of the underground articulated scraper is obtained as following [42]:

(17) $$\left\{\begin{array}{c} \dot{\varepsilon_{1}}\\ {\dot{\varepsilon }}_{2}\\ \dot{\varepsilon_{3}} \end{array}\right\}=\left[\begin{array}{ccc} 0 & v & 0\\ 0 & 0 & v\\ 0 & 0 & 0 \end{array}\right]\left\{\begin{array}{c} \varepsilon_{1}\\ \varepsilon_{2}\\ \varepsilon_{3} \end{array}\right\}+\left\{\begin{array}{c} 0\\ \left(\frac{L_{2}}{L}\right)\\ \left(\frac{1}{L}\right) \end{array}\right\}\;\dot{\gamma }.$$

The difference between homeopathic heading angle $\theta_{f}$ and expected heading angle $\theta_{i}$ was defined as heading angle deviation $\varepsilon_{\theta }$. The distance between the reference location point P and the expected reference location point $P_{i}$ of the underground scraper is taken as the transverse deviation $\varepsilon_{d}$ (positive when the reference location point P is on the right side of the expected path): the curvature deviation between the reference location point P and the expected reference location point $P_{i}$ is $\varepsilon_{c}$.

Under the condition of uniform speed, the dynamic model of the deviation of the articulated scraper is a linear time-invariant system model, and the articulated angle can be controlled by controlling the error variable.

### 2.2. Path Tracking of Underground Articulated LHD Based on LQR Controller

The control object of LQR optimal controller is a linear system expressed by state space and other basic structures in modern control theory, and all state variables of the system are required to be fully controllable and observable [43]. The core concept of LQR control is to achieve the maximum control effect with the minimum control variable, that is, the minimum energy consumption [44]. The optimal state design stage of LQR refers to the K design of diverse state feedback microcontrollers required in the optimization stage, which requires that we can simultaneously make the two objective vector functions of quadratic type Q and R take the lowest value. The state feedback matrix K of the target is the unique determination of Q and R matrices in LQR control, and both Q and R matrices are positive definite matrices [45].

According to the LQR control theory, the state-space equation of the controlled object requires being determined first. The state equation of the underground articulated scraper can be set as the following equations [46]:

(18) $$\{\begin{array}{c} \dot{X}\left(t\right)=A_{1}X\left(t\right)+B_{1}u\left(t\right)\\ Y\left(t\right)=C_{1}X\left(t\right)+D_{1}u\left(t\right) \end{array}.$$

Note: $A_{1}$ is the system state space, $A_{1}\in R^{n\times n}$, and *R* is the n-dimensional real matrix. $B_{1}$ is the input vector of the system, $B_{1}\in R^{n\times 1}$, and *R* is the n-dimensional vector. $C_{1}$ is the system output matrix, $C_{1}\in R^{n\times n}$, and R is the n-dimensional real matrix. $D_{1}$ is the state feedback vector, $D_{1}\in R^{n\times 1}$, and *R* is the n-dimensional real vector. X(t), u(t), Y(t) are system input variables, state variables, and outputs.

According to the above mathematical model of the scraper and vehicle parameters of the articulated scraper in Table 1, the parameters of the state-space equation of the underground articulated scraper can be determined as following [47]:

(19) $$A_{1}=\left[\begin{array}{ccc} 0 & 3.5 & 0\\ 0 & 0 & 3.5\\ 0 & 0 & 0 \end{array}\right]\cdot B_{1}=\left[\begin{array}{c} 0\\ \frac{3.44}{5.12}\\ \frac{1}{5.12} \end{array}\right]\cdot C_{1}=\left[\begin{array}{ccc} 1 & 0 & 0\\ 0 & 1 & 0\\ 0 & 0 & 1 \end{array}\right].$$

According to the above matrix parameters, the energy control and visual analysis of the system are:

(20) $$\{\begin{array}{c} rank\left[\begin{array}{ccc} B & AB & A^{2}B \end{array}\right]=3\\ rank\left[\begin{array}{ccc} C & CA & CA^{2} \end{array}\right]=3 \end{array}.$$

From the above equation, it can be seen that the observability and the controllability matrix of the system are full rank, which means that the underground articulated scraper system is fully controllable and observable, meeting the basic conditions of LQR control [48]. The state space equation model of the system can be determined by the deviation dynamics model and parameters of the underground articulated LHD:

(21) $$\{\begin{array}{c} \dot{\varepsilon }\left(t\right)=A\varepsilon \left(t\right)+B\dot{\gamma }\left(t\right)\\ Y\left(t\right)=\varepsilon \left(t\right) \end{array}.$$

Through the output deviation matrix $\varepsilon \left(t\right)=\left[\begin{array}{ccc} \varepsilon_{1} & \varepsilon_{2} & \varepsilon_{3} \end{array}\right]$ of the control system, the ideal articulation angle input of the car body can be obtained, and the optimal control performance index can be established [49].

(22) $$J\left(\dot{\gamma }\right)=J_{0}\left(t\right)+J_{1}\left(t\right)=\int_{0}^{\infty }\left[\varepsilon^{T}\left(t\right)q\varepsilon \left(t\right)+{\dot{\gamma }}^{T}\left(t\right)r\dot{\gamma }\left(t\right)\right]dt$$

Note: $J_{0}$ is the time domain integral of the deviation of the underground articulated scraper is the error performance index. $J_{1}$ represents the time domain integral of control quantity, namely the energy consumption index.

The core of LQR control is to achieve the best control effect with the minimum error performance index and the minimum energy consumption index, establish the feedback control rate u=−KX(t) to achieve $K=R^{-1}B^{T}P$, and establish the Riccati equation:

(23) $$A^{T}P+PA+Q-PBR^{-1}B^{T}P=0.$$

Note: *P* is some definite positive matrix, and let P=E, *E* is the identity matrix.

**Figure 5:**
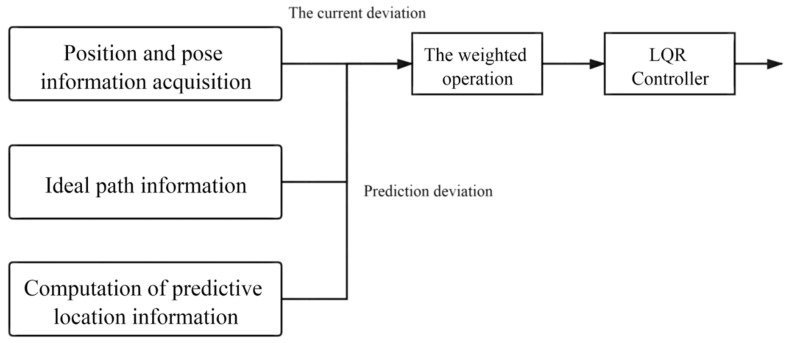
LQR parameter configuration process.

To achieve the best input of the control system, it is necessary to reasonably configure the *Q* and *R* matrices to achieve the ideal output of the control quantity [50]. The configuration process of *Q* and *R* parameters is shown in Figure 5.

The input of the LQR controller is composed of three parts. One is the acquisition of the position and posture information of the scraper related to the current position and the input of the LQR controller [51]. Secondly, the acquisition of ideal path information means that the input of LQR controller is related to the position information of the ideal path. The third is the collection of predictive position information, which is solved by using the deviation dynamics model [52]. Therefore, the controller input of LQR should be a linear superposition of the above three variables. In order to prevent overcontrol and under-control of the underground articulated LHD, different weights should be given to the three variables after comprehensive consideration. The strategy adopted in Part I is to obtain the difference between the current position information and the ideal path, and the predicted position information is to obtain the error by calculating the deviation dynamics model. The final input of the LQR controller is the weighted superposition of the two, as shown in Equation (24).

(24) $$\{\begin{array}{c} \varepsilon \left(t\right)=a\varepsilon_{a}\left(t\right)+b\varepsilon_{b}\left(t\right)\\ \varepsilon_{a}\left(t\right)=\left[\begin{array}{ccc} \varepsilon_{1} & \varepsilon_{2} & \varepsilon_{3} \end{array}\right]\\ \varepsilon_{b}\left(t\right)=\left[\begin{array}{ccc} \varepsilon_{1}{}^{\prime } & \varepsilon_{2}{}^{\prime } & \varepsilon_{3}{}^{\prime } \end{array}\right] \end{array}$$

Note: $\varepsilon_{1}\left(t\right)$ is the Current Tracking Deviation Matrix; $\varepsilon_{2}\left(t\right)$ is the Prediction Information Deviation Matrix; *a* and *b* are the weight factors, *a* + *b* = 1; ε(t) is the final input for the LQR controller.

### 2.3. Algorithms

The state variable weight matrix Q in the LQR control can control the amount of weight matrix R and then use that to determine the state feedback vector. The selection of matrix Q and R parameters will directly relate to the effects of control. According to the parameter-setting problem in the classical control theory, the selection of two positive matrices Q and R are prone to rely on a grand amount of engineering experience, which takes longer [53]. Moreover, the optimal parameter configuration of Q and R cannot be obtained. The selection of the weight matrix Q of the state variable and the weight matrix R of the control quantity can be simply regarded as the Travel Salesperson Problem. It is difficult to obtain the optimal solution for such problems with general methods, so we require solving them with the help of some enlightening intelligent clustering algorithms, such as genetic algorithm (GA), Ant Colony Algorithm (ACA), and Micro Particle Swarm Optimization (PSO) [54].

#### 2.3.1. Adaptive GA Algorithm Optimization

(1) Disadvantages of simple genetic algorithms

The simple genetic algorithm (SGA) is of great significance in practical engineering applications, but nowadays, many defects of the classical simple genetic algorithm are exposed in the process of engineering practice, such as “population precocity”, population differentiation and various groups still do not show the identity after various choices, and so on [55]. The unreasonable structure of natural selection, crossover, and the mutation algorithm is the fundamental reason for the precocity problem of the population. The precocity problem cannot be avoided, which is also a major feature of an intelligent clustering algorithm. Therefore, it is necessary to improve the crossover operator and mutation operator of the classical genetic algorithm to solve the problem of population precocity to some extent [56].

(2) Improved adaptive genetic algorithm LQR control (LQR–AGA)

In LQR control, the improved adaptive genetic algorithm, as an improved intelligent clustering algorithm, conquers the shortcomings of traditional LQR control. The parameter selection of *Q* and *R* is optimized by the population, and the crossover mutation operator of the improved genetic algorithm has a strong global optimization ability, and it can find the best state feedback matrix in the selected space [57].

**Figure 6:**
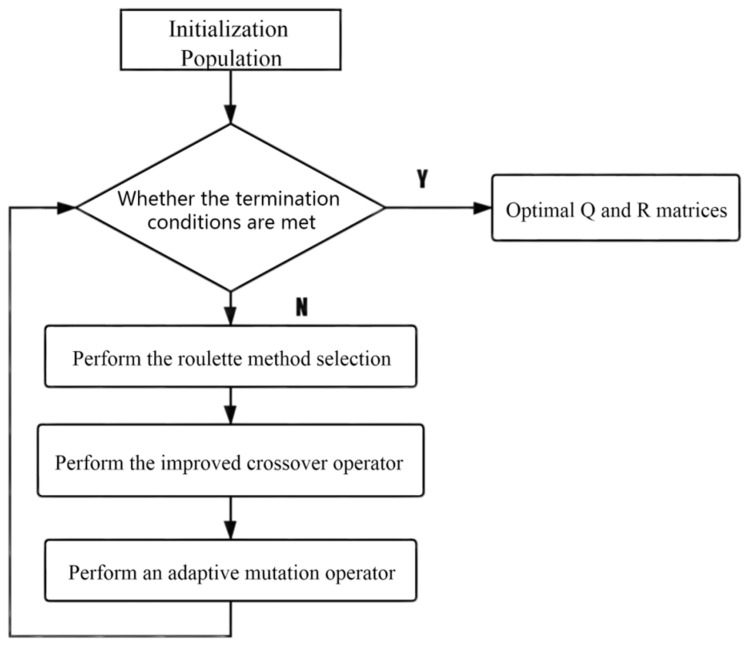
AGA algorithm flow chart.

The improved adaptive genetic algorithm process is shown in Figure 6, whose main function is to optimize the *Q*, *R* two matrices in the LQR controller, that is, to determine the parameters of the optimal $Q=\left[\begin{array}{ccc} q_{1} & 0 & 0\\ 0 & q_{2} & 0\\ 0 & 0 & q_{3} \end{array}\right]$ and *R* matrix.

##### Encoding

The improved adaptive genetic algorithm can encode chromosomes and genes by using the real encoding method because the real encoding method is intuitive, simple, and easy to calculate [58]. This method is suitable for the calculation of genetic algorithms with complex fitness function and can greatly diminish the calculation amount of genetic algorithms, so as to speed up the running efficiency of the genetic algorithm [59].

For example, the chromosome is assumed to be $Q=\left[\begin{array}{ccc} q_{1} & 0 & 0\\ 0 & q_{2} & 0\\ 0 & 0 & q_{3} \end{array}\right]$; of these, *q*_1_, *q*_2_, and *q*_3_ are genes on the chromosome, while *Q* is the operation of selection, crossover, and mutation operators after the participation of chromosomes and individuals.

##### Group Value Range

Chromosome *Q* is generated according to the MATLAB random number matrix; that is, the initial trial of the population has a strong randomness, which expands the global optimization ability of the improved adaptive genetic algorithm.

(25) $$Q=\left[\begin{array}{ccc} q_{1} & 0 & 0\\ 0 & q_{2} & 0\\ 0 & 0 & q_{3} \end{array}\right]=\left[\begin{array}{ccc} 50*rand & 0 & 0\\ 0 & 50*rand & 0\\ 0 & 0 & 50*rand \end{array}\right]$$

It can be seen from Equation (25) that $q_{1}\in \left[0,50\right]$, $q_{2}\in \left[0,50\right]$, $q_{3}\in \left[0,50\right]$, the stability of the whole system and the state feedback matrix must exist, which can be guaranteed by such a value [60].

##### Interleaved Mode

The improved crossover operator is used to select the parent generation for crossover change to produce the offspring with strong search ability.

(26) $$\{\begin{array}{c} y_{i}^{\left(1\right)}=a\left(x_{i}^{\left(1\right)}-x_{i}^{\left(2\right)}\right)+bx_{i}^{\left(1\right)}+cx_{i}^{\left(2\right)}\\ y_{i}^{\left(2\right)}=a\left(x_{i}^{\left(1\right)}-x_{i}^{\left(2\right)}\right)+bx_{i}^{\left(2\right)}+cx_{i}^{\left(1\right)} \end{array}$$

(27) $$\{\begin{array}{c} y_{i}^{\left(1\right)}=bx_{i}^{\left(1\right)}+cx_{i}^{\left(2\right)}\\ y_{i}^{\left(2\right)}=bx_{i}^{\left(2\right)}+cx_{i}^{\left(1\right)} \end{array}$$

Note: $x_{i}^{\left(n\right)}$ is the n gene above the parent line *i* chromosome; $y_{i}^{\left(n\right)}$ is the n gene above the offspring clause *i* chromosome; a, *b*, and *c* represent the cross variants.

The two crossover modes are selected according to whether the children cross the boundary or not. If the children cross the boundary, they cross according to Equation (26); otherwise, they cross in accordance with Equation (27) [61].

##### Variation

According to the adaptive mutation operator, the random gene location on the chromosome was mutated to ensure the diversity of the population and enhance its global optimization ability. Meanwhile, it also ensured that the population could have the identity and converge to the optimal solution in the later iteration period.

(28) $$\Omega =\left\{x_{k}-s\left(t\right)\times \left(x_{k}-L_{k}\right),x_{k}+s\left(t\right)\times \left(U_{k}-x_{k}\right)\right\}$$

Note: $s\left(t\right)=1-C^{{\left[1-\left(\frac{1}{G}\right)\right]}^{k}}$; Ω represents size range of the variation action; $x_{k}$ represents genes; $L_{k}$ represents the minimum range of variation of the previous generation; $U_{k}$ represents the maximum range of variation of the previous generation; s(t) represents algebraic variants.

When the number of iterations is small, the gene probability is large, and the global optimization ability of the population is strong. When the number of iterations is high, the mutation probability is small, the computational speed of the genetic algorithm is high, and the required time is short [62].

##### Parameter Selection

The number of initialized population individuals was set to be 30, and 50 generations were bred. The probability of crossover between two chromosomes was 0.2, the variation action constant *b* = 3, and the range of population living space was [0, 50].

Through $J\left(\dot{\gamma }\right)$ discretization of LQR, the fitness equation can be obtained as follows:

(29) $$F_{\left(i\right)}=\sum_{t=1}^{T}q_{1}\varepsilon_{1}{}^{2}\left(t\right)+q_{2}\varepsilon_{2}{}^{2}\left(t\right)+q_{2}\varepsilon_{3}{}^{2}\left(t\right)+{\dot{\gamma }}^{2}\left(t\right).$$

Note: *T* represents the total sampling time length; *q*_1_, *q*_2_, and *q*_3_ represent the Q matrix diagonal elements.

**Figure 7:**
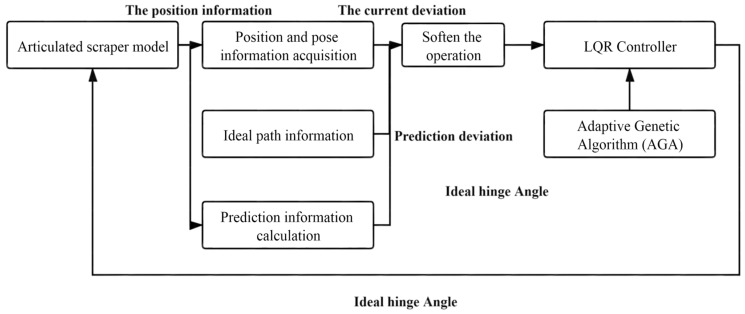
LQR-AGA control system block diagram.

To sum up, the block diagram of the LQR-AGA control system can be drawn, as shown in Figure 7 [63].

(3) Simulation experiment of LQR-AGA control algorithm

**Figure 8:**
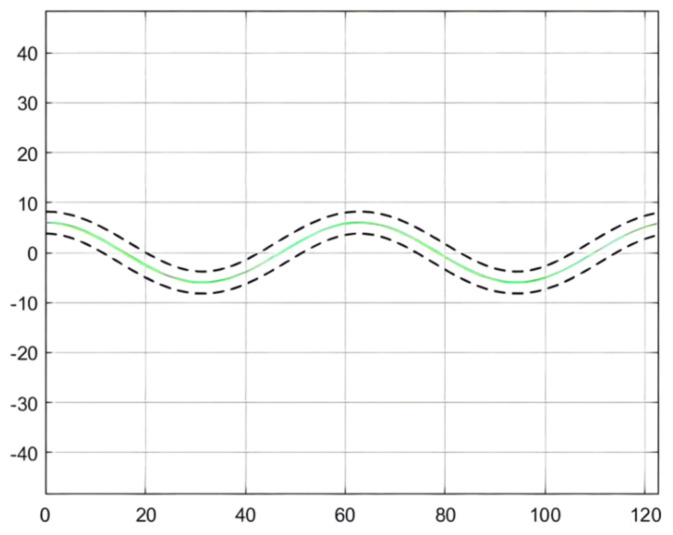
Simulation path of an underground articulated scraper.

For the simulation of the path, Figure 8 shows the wavy roadway and the halfway point of the cross-section of the roadway in the attachment for the ideal scraper run path, which controls the target path. This path has continuous turning and other complex road conditions, so the controller detection needs to have strict conditions to embody the scraper movement in actual operation [64]. In addition, in order to ensure the safe operation of the scraper, the maximum lateral deviation, namely, the safe distance, should be set within 0.6 m [65].

For example, it is presumed that the population size N is set to 30 and the number of iterations G can be set to 50 generations. The probability of crossover is about 0.2, which can ensure that the population has strong adaptability and can have a better global optimization ability and algorithm iteration speed. The adaptive variation constant 0.2 can ensure that the population has a relatively good global optimization ability and avoid falling into local optimization in the iteration. The variation action constant b = 3 guarantees the global capability at the initial stage of variation and ensures that the local optimum will not fall into at the end of variation. The survival range of the population [0, 50] ensures the positive nature of the control matrix and the stability of the whole control system [66].

In summary, all the parameters of LQR-AGA are set as shown in Table 2 below.

The initial test coordinate of the articulated scraper is set as [0.00, 6.50], the initial test heading angle is set as 0π, the hinged steering angle is returned to zero, and the driving speed is constant at 3.5 m/s. The AGA algorithm is used to optimize the weighted matrix in the path tracking controller of the articulated scraper. After repeated experiments, we found that in the first 20 iterations of the AGA algorithm, the population has already had strong spatial distribution and global search ability. The spatial distribution and variation are wide. In the last 20 generations, the population shows strong convergence and quickly converges to the optimal solution of the living space. The adaptations of whole populations to humans and other creatures in nature are the same as the adaptations of humans to populations [67]. Table 3 shows the results of parameter optimization. The results reveal that other parameters have the optimal adaptability in the iterative time environment. In the 50 iterations, the fitness of the AGA algorithm showed monotonically soaring, indicating that both the individuals and the population were evolving toward the position of the optimal solution, and the fitness of the population remained stable at the end of the iteration, indicating that the entire population had converged to the optimal solution [68].

**Figure 9:**
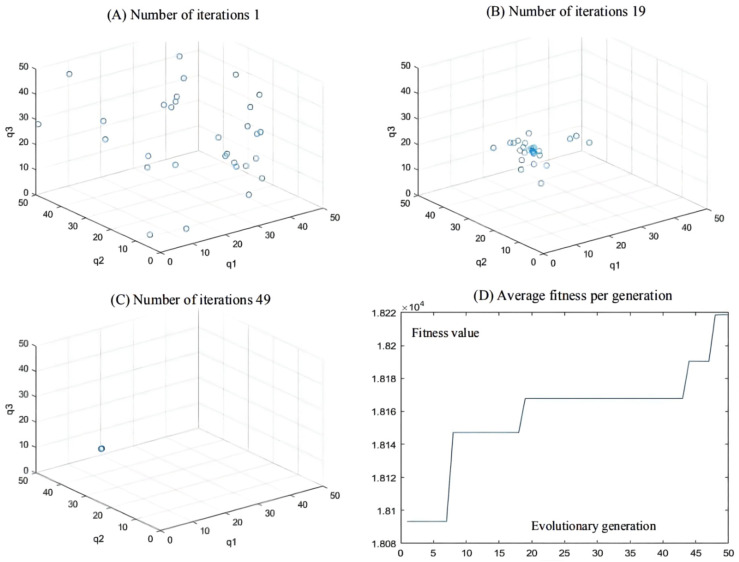
(**A**–**C**) Population distribution of the 1st, 19th, and 49th generations; (**D**) Average fitness per generation.

**Figure 10:**
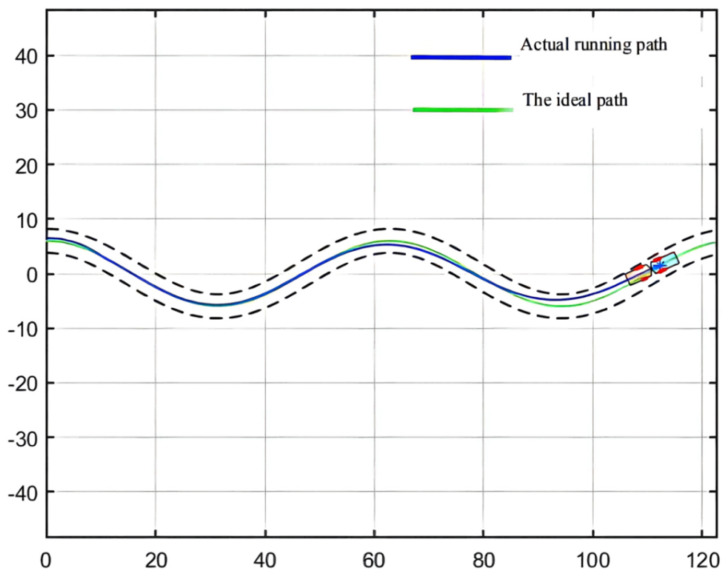
Simulation result.

**Figure 11:**
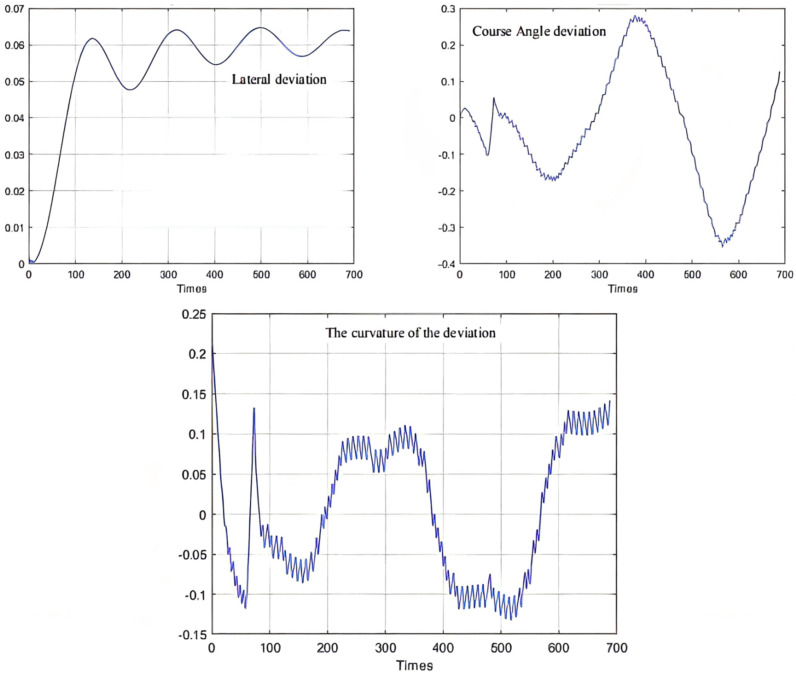
Deviation range.

As can be seen from Figure 9, Figure 10 and Figure 11, the population has a strong global optimization ability at the beginning, the convergence rate is fast at the later stages of iteration, and the average fitness of the population is high at the end of the iteration. From the perspective of the simulation environment, the lateral error of the LHD on the simulated path is less than 0.1 m, so it can be seen that the weighted matrix Q optimized by the AGA algorithm makes the actual route of the articulated LHD coincide with the ideal path [69].

#### 2.3.2. Optimization of QPSO Algorithm

(1) Disadvantages of simple PSO algorithm

The model of the simple PSO algorithm is the BOID (bird-oid) model of birds’ predation behavior, which simulates the predation characteristics of gregarious creatures. Due to its low requirement for the objective function, simple programming, and easy programming, this model algorithm plays an important role in data scheduling, optimization processing, function optimization analysis, intelligent training and neural network, and other emerging disciplines. However, the BOID model also has obvious disadvantages, such as the severe precocity problem of the population, the strong randomness of the optimization results, and that the global optimal advantage can only be found when the number of iterations approaches infinity. These reasons will result in that the PSO algorithm of the BOID model cannot satisfy the parameter optimization function of the LQR controller, because the objective function of the LQR is a complex multi-peak function, the randomness of solving it by the BOID model is too large, and it cannot guarantee that the optimization results can meet the path tracking requirements of the underground articulated scraper. Therefore, it is necessary to improve the simple PSO algorithm to achieve the optimization ability of an LQR objective function [70].

(2) Quantum Behavior PSO Algorithm (QPSO)

This particle swarm optimization algorithm strengthens the global optimization ability of each individual in the population. Combined with the linear weight reduction strategy, the inertia of the individual group can be reduced at the end of the iteration to accelerate its convergence rate and accelerate the group searching efficiency. The optimized particle swarm speed iterative algorithm is shown in Equation (30):

(30) $$\{\begin{array}{c} V_{i}=wV_{i}+a_{1}rand()\left(Pbest-X_{i}\right)+a_{2}rand()\left(Gbest-X_{i}\right)\\ X_{i}=X_{i}+V_{i}\\ W=\left(W_{ini}-W_{end}\right)\times \frac{G_{k}-g}{G_{k}}+W_{end} \end{array}.$$

Note: $V_{i}$ represents the velocity of the *i*-th particle; $X_{i}$ represents the location of the *i*-th particle; *Pbest* represents the historical optimal position of the particle; and *Gbest* represents the historical optimal location of the population.

In view of the particle swarm optimization algorithm (PSO), in the late iteration of high dimension, it is easy to fall into local optimum and other problems. Therefore, the concept of the hand velocity factor needs to be introduced to increase the velocity of particles at the end of the iteration. Its formula is shown in Equation (31):

(31) $$V_{i}=K\;[V_{i}+a_{1}rand\left(\;\right)\times \left(P_{i}-V_{i}\right)+a_{2}Rand()\times \left(P_{g}-X_{i}\right).$$

In the early stage of the algorithm iteration, because the particle distribution is relatively scattered and the particle has a large inertia weight at this time, it will explore the space globally according to its initial velocity, and the particle at this time has a strong global exploration ability. Therefore, the *K* values should be large initially. In the late iteration of the algorithm, the population needs to have strong convergence characteristics, so as to speed up the operation speed of the algorithm, and the population requires changing in a small spatial range. To sum up, *K* values should show a monotonically decreasing characteristic with the increase in the number of iterations, so we can set the function of K values changing with the number of iterations as shown in Equation (32).

(32) $$K=\frac{\cos(\left(\frac{T}{\max\_Gen}\right)T)+2.5}{4}$$

Note: *T* represents number of current iterations; Max_*Gen* represents the maximum number of iterations.

QPSO adjusts the position update strategy in the simple PSO algorithm by canceling the attribute of speed and replacing it with the probability distribution function, which means that particles are distributed according to probability rather than velocity. Therefore, the spatial attribute of each particle in the population needs to be determined by every observation. The formula for calculating the average value of the historical optimal fitness of a single particle is as follows:

(33) $$m=\frac{1}{N}\sum_{i}^{N}pest_{i}.$$

Note: *N* is the sum of the number of particles; $Pest_{i}$ represents the optimal fitness of a single particle in the *i*-th iteration.

The position update of particles is based on the probability distribution function, as shown in Equation (34):

(34) $$\{\begin{array}{c} P_{i}=\xi \times pest_{i}+\left(1-\xi \right)\times gest_{i}\\ X_{i}=P_{i}\pm a\left|m-X_{i}\right|\times ln\left(\frac{1}{b}\right) \end{array}.$$

Note: ξ represents the probability function obeying the uniform distribution on (0,1); ±α represents the expansion coefficient, the probability of positive is 50%, and the probability of negative is 50%.

**Figure 12:**
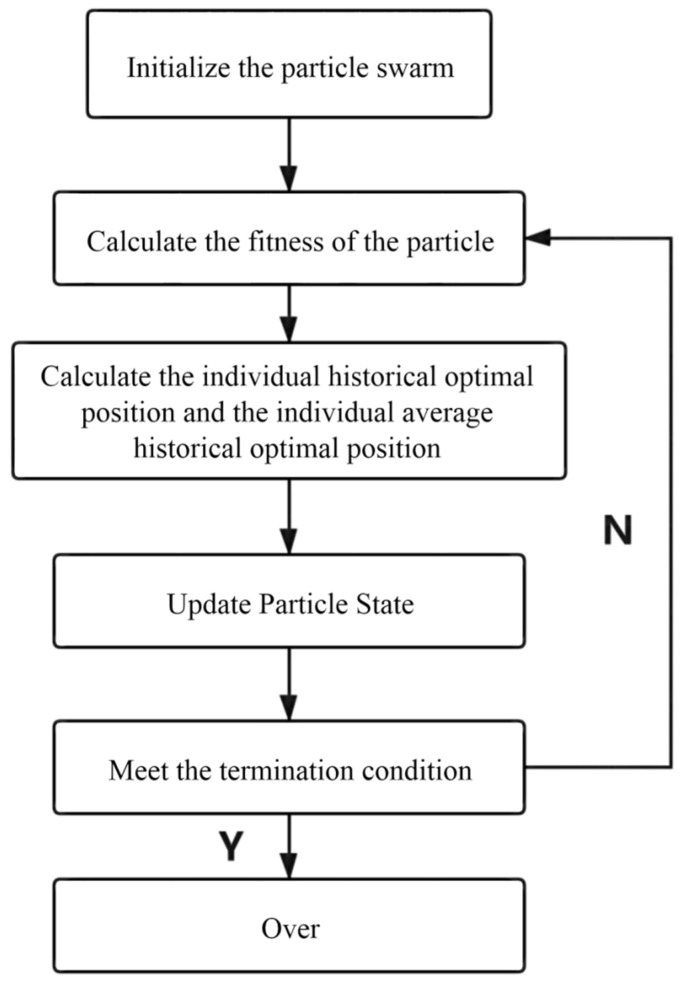
Particle group algorithm flow.

**Figure 13:**
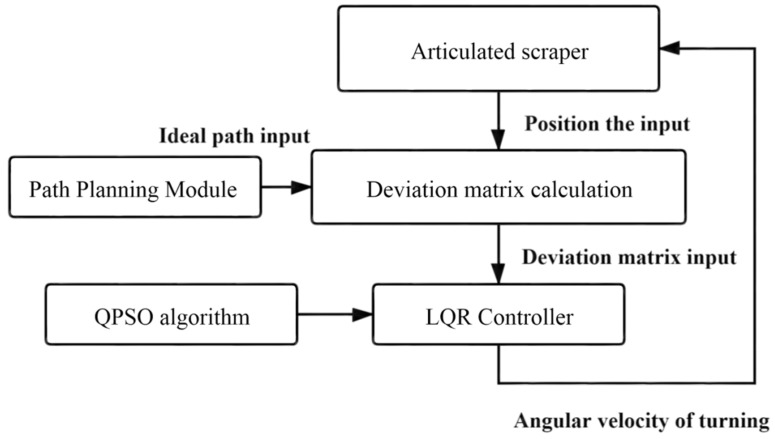
Particle group algorithm flow.

The particle swarm optimization analysis algorithm directly uses a particle group; each particle in the individual information is used to group the comprehensive analysis and information sharing of the information to directly promote the coordinated motion of particle groups, so that it will directly produce the evolution process from disorder to order in space in the process of solving a population problem. Thus, we can directly obtain the optimization and understanding of a group problem whose basic flow A and block diagram B of the LQR-QPSO system are shown in Figure 12 and Figure 13.

(3) LQR-QPSO control algorithm simulation experiment

The simulation environment is shown in Figure 8; see Table 1 for the body parameters of the underground articulated scraper; the QPSO parameter configuration is shown in Table 4. The maximum lateral deviation is required to be less than 0.6 m [71].

**Figure 14:**
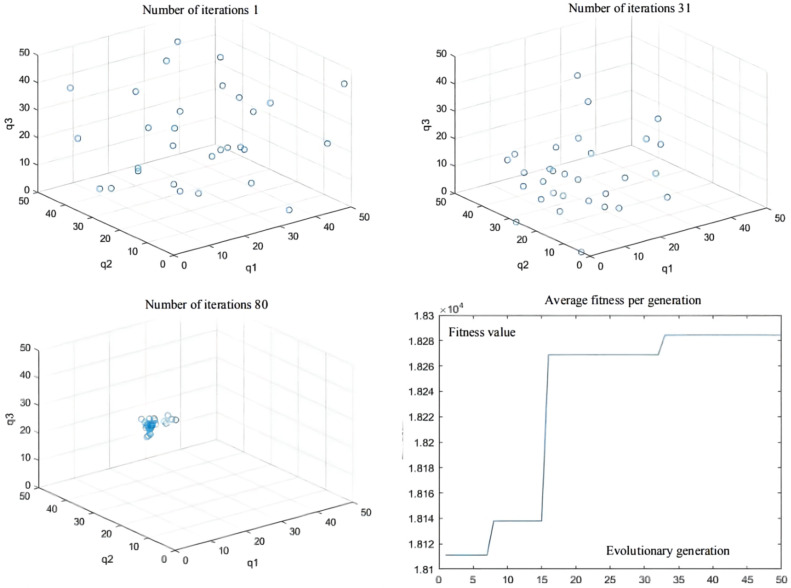
Population distribution of the 1st, 31th, and 80th generations; and average fitness per generation.

The initial test coordinate of the articulated scraper is set as [0.00, 6.50], and the heading angle of the initial test is set as zero angle, namely, 0°. In addition, the steering angle of the front and rear car bodies of the articulated scraper is set to zero, which means that the body keeps moving forward, and the traveling speed is 3.5 m/s, so it is inconvenient to maintain the speed constant. After repeated trial and simulation tests, it is found that in all experiments, the population convergence rate of the QPSO algorithm is slow. In the first 30 generations, each particle varies greatly in the global scope, showing obvious divergence, but after 60 generations, particle swarm gradually converges to the optimal value. The controller parameters obtained in the 80 generations can make errors in the operation process of the articulated scraper within a reasonable range [72], as shown in Figure 14.

**Figure 15:**
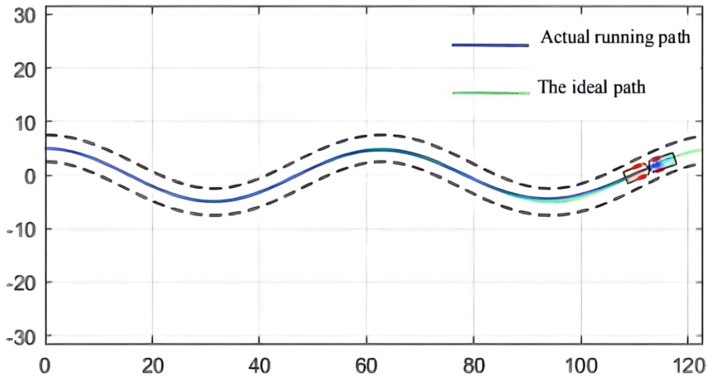
Simulation results.

**Figure 16:**
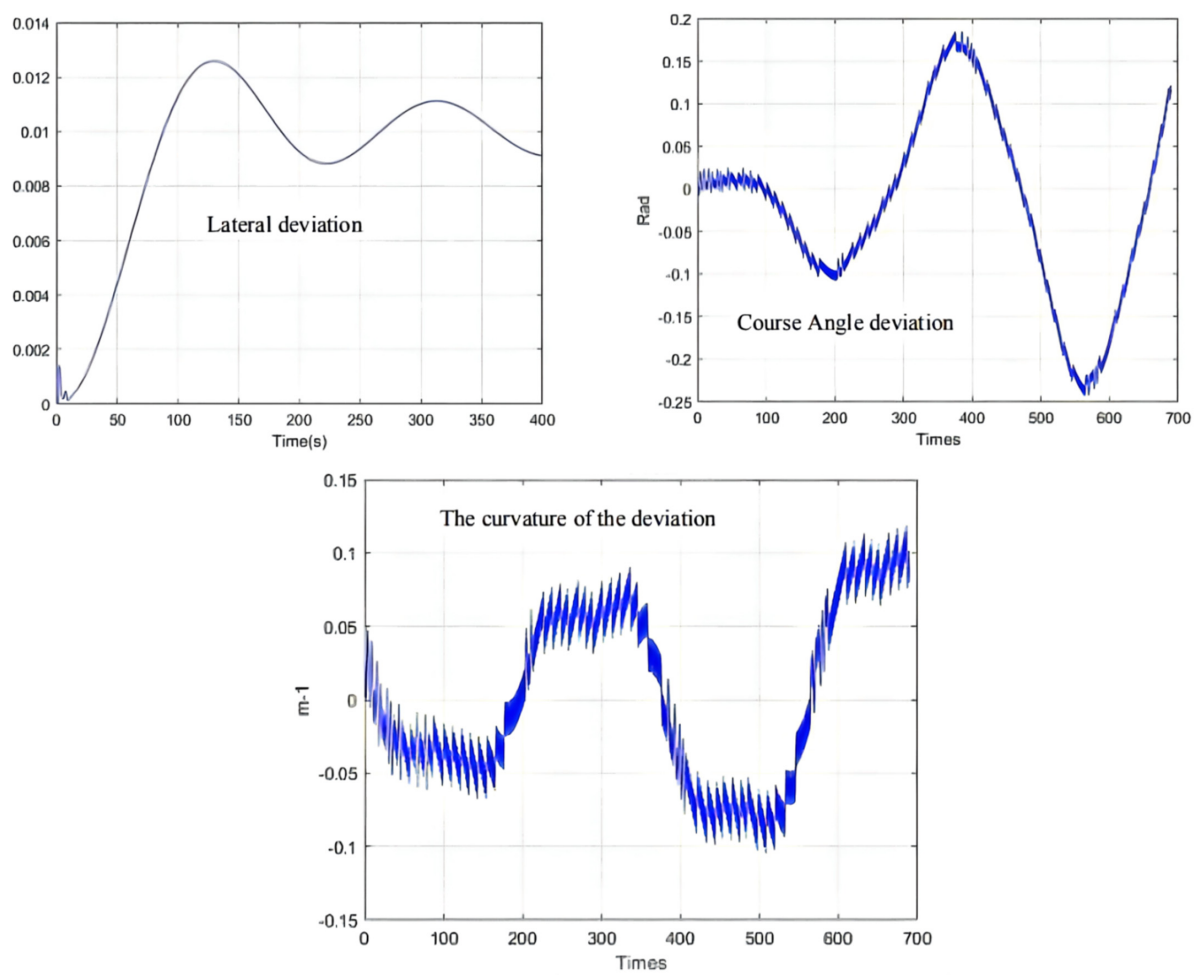
Deviation range.

Besides, the optimization results of the QPSO algorithm are shown in Table 5, the simulation results of QPSO are shown in Figure 15, and the deviation range of QPSO is shown in Figure 16.

#### 2.3.3. ACA Optimization of Ant Colony Algorithm

(1) Ant Colony Algorithm LQR Controller (LQR-ACA)

**Figure 17:**
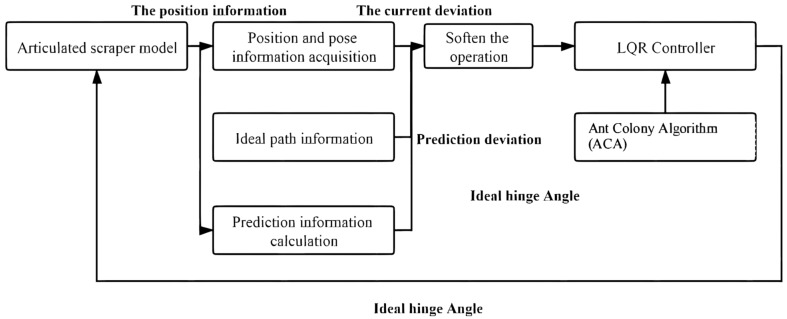
LQR-ACA control system block diagram.

An LQR-ACA path tracking controller can be established as shown in Figure 17 [73].

(2) LQR-ACA control algorithm simulation experiment

The validity and reliability of the LQR-ACA path traceability controller were tested and verified by MATLAB simulation [74]. To make this ant group have better search ability and code iteration speed, the ant number is set as 30, and the search time G is set as 50 generations. The hormone play factor is set to 0.4, and the search range is set to (0, 50), [75]. The ACA parameter configuration is shown in Table 6.

**Figure 18:**
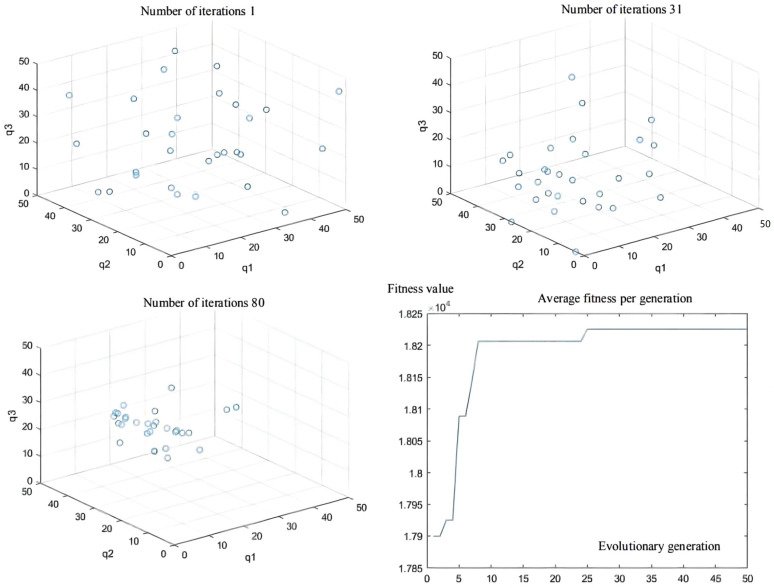
Population distribution of the 1st, 31th, and 80th generations; and average fitness per generation.

The initial coordinate of the articulated scraper is set as [0.00, 6.50], the heading angle of the initial test is set as 0 π, the hinged steering angle is returned to zero, and the driving speed is constant at 3.5 m/s. The ant colony algorithm is used to configure the parameters of the LQR controller of the articulated scraper. After testing repeatedly, it is found that the ant colony algorithm of an ant colony in 100 iterations will converge to different extreme value points, and the position of most of the ants in the number of iterations is more than 20. Since it no longer changes generations, the fitness function of the LQR controller is a more extreme value point function, and there were many equal fitness points in the solution space [76], as shown in Figure 18.

Besides, ACA algorithm optimization results are shown in Table 7.

**Figure 19:**
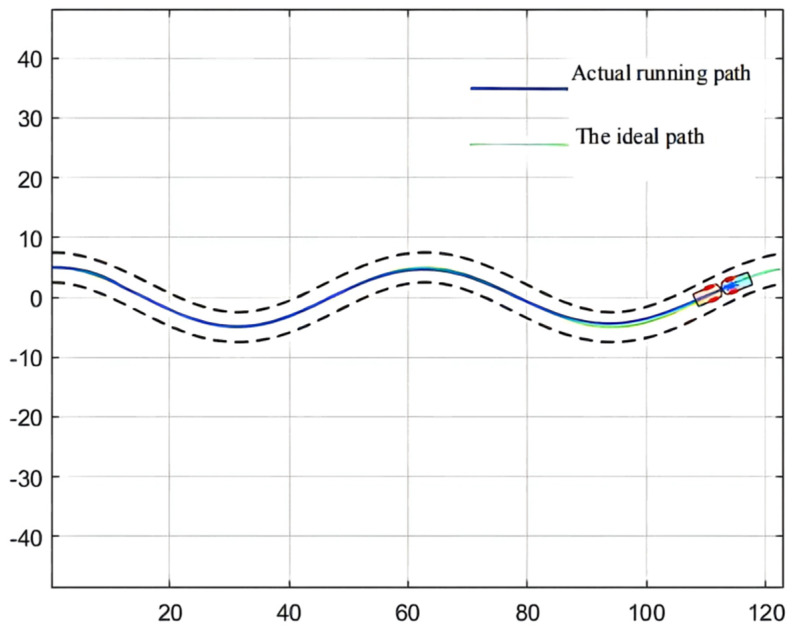
Simulation results.

**Figure 20:**
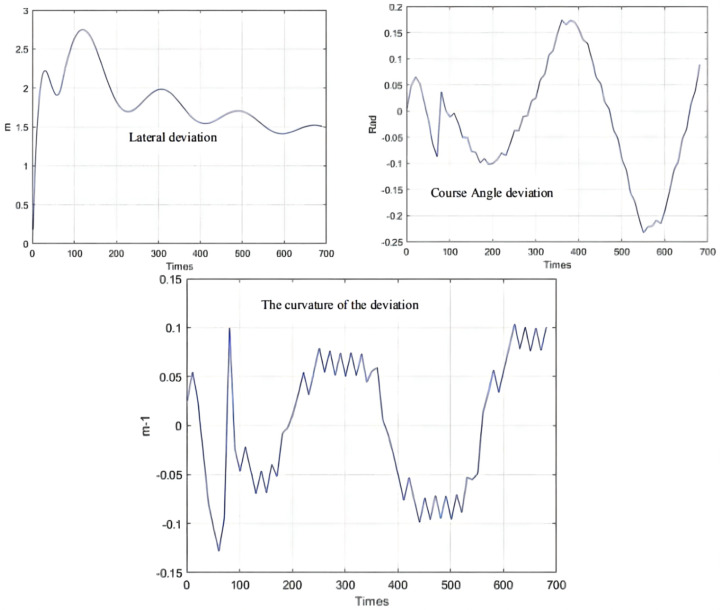
Deviation range.

The parameter optimization results are brought into the simulation environment of the articulated scraper to complete the path tracking simulation, and the results as shown in Figure 19 can be obtained. The weighted matrix Q obtained by the ACA algorithm makes the actual route of the articulated scraper coincide with the ideal path [77]. Besides, the deviation range of ACA is shown in Figure 20.

## 3. Results

### 3.1. Comparison of Algorithm Parameter Configuration

The population size of AGA, QPSO, and ACA was 30. Both the AGA and QPSO populations converge to a certain extreme point, but the AGA population converges faster than QPSO, while the ACA population converges to multiple extreme points, and the calculation time is longer. The parameter configuration comparison is shown in Table 8.

### 3.2. Comparison of Algorithm Results

The parameter configuration of the Q matrix obtained by the three intelligent clustering algorithms is intensely different, but they all have high fitness. AGA had the highest fitness, while ACA had the lowest fitness. The comparison of algorithm optimization results is shown in Table 9.

### 3.3. Comparison of Simulation Results

**Figure 21:**
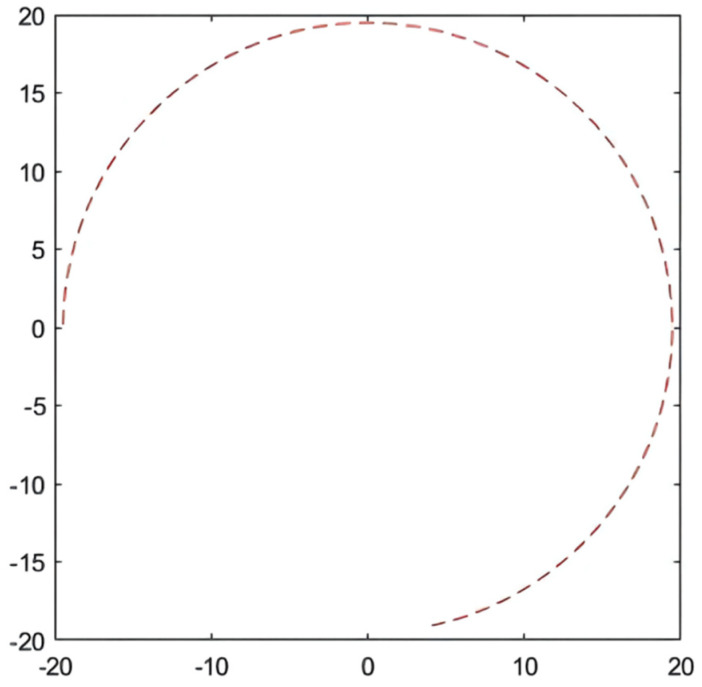
Contrast experimental simulation path.

**Figure 22:**
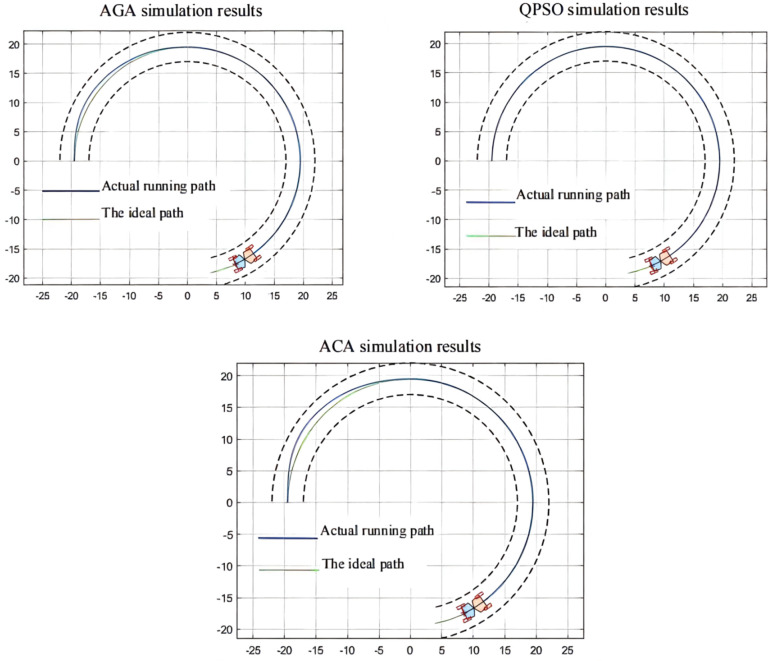
Comparison simulation results.

In the simulation environment, the ideal path of the articulated scraper is a circular trajectory with (0, 0) as the center of the circle and a radius of 5, as shown in Figure 21. It can be seen from Figure 22 that the optimization results of the three clustering algorithms can all meet the error requirements. In the simulation, the initial error of the optimization results obtained by the AGA algorithm and the ACA algorithm is large. After about a quarter of a semicircular trajectory, the optimization controllers obtained by the AGA and ACA intelligent clustering algorithms can reduce the deviation degree between the LHD and the ideal path in the subsequent operation through the control effect. The optimization results obtained by the QPSO algorithm are always less deviated from the ideal path.

## 4. Discussion

In our study, AGA, QPSO, and ACA are intelligent cluster algorithms that can all seek optimal results for LQR parameter configuration problems. We did not clearly indicate which algorithm (AGA, OPSO, or ACA) is better, because each of them has its own advantages and disadvantages and will have different performance in specific different situations. Through the simulation, we found the advantages and disadvantages of these three algorithms (AGA, OPSO, or ACA), and our study can definitely provide a reference for the future researchers:

(1) The QPSO algorithm has slow operation speed and slow group convergence speed, but it can find the optimal solution;
(2) The AGA algorithm has fast operation speed and fast group convergence, but the optimization result is poor compared with the QPSO algorithm;
(3) The ACA algorithm has slow operation speed and slow population convergence speed, but it can converge to multiple extreme points and has a large space for optimization.

However, there are some problems that are reflected in our research:

(1) Firstly, the biggest characteristic of the intelligent cluster algorithms is the problem of premature data; in order to solve the problem, the development direction of this kind of algorithm is to improve the intelligent cluster algorithm;
(2) Secondly, in terms of LQR parameter configuration, the fitness function is the main reason for the slow operation speed of the intelligent cluster algorithm; to solve the problem of operation speed, it is necessary to simplify and redefine the fitness function of LQR to reduce the operation time.

Therefore, in order to completely realize the unmanned driving of LHD, our researchers need to continue efforts from the following two points:

(1) Optimize the controller itself.

As early as in 2010, C. W. Tao, J. S. Taur, and Y. C. Chen [78] compared the advantages and disadvantages of Fuzzy controller and LQR controller. They thought that Fuzzy controller and LQR controller may be suitable for different situations, but they still discussed the possibility of combining LQR controller and Fuzzy controller.

In 2019, Y. I. Kudinov et al. [79] proposed that the Fuzzy FLC+LQR controller has a much higher speed than the LQR controller, and in 2020, Z. B. Hazem, M. J. Fotuhi, and Z. Bingül [80] developed F-LQR (Fuzzy LQR) controller and F-LQG (Fuzzy LQG) controller and applied them to the stability control of a two-link rotary inverted pendulum.

Thus, in our future work, we will do the further research to investigate the applicability of other controllers, such as Fuzzy LQR, for a driver-less scraper (LHD).

(2) Algorithm optimization.

Although in our study we only compared the simulation effects of the AGA, QPSO, and ACA algorithms, F. Amini, N. K. Hazaveh, and A. A. Rad [81] used the discrete wavelet transform (DWT) algorithm and H. Wang et al. [82] used the artificial bee colony (ABC) algorithm to do similar research around 2014.

Thus, in the future, we will try to compare the advantages and disadvantages of more algorithms and then optimize the algorithm according to the different situations.

## 5. Conclusions

This paper studies the path tracking control of a driver-less scraper in the underground mine, concentrating on the linear quadratic optimal control (LQR) and the parameter optimization of three intelligent clustering algorithms: AGA, QPSO, and ACA. The main work is summarized as the following:

(1) For articulated LHD path tracking control problems, we studied the kinematics model of the articulated LHD body through the analysis of the kinematics modeling, determining the articulated LHD vehicle reference speed of the anchor point, course angular velocity, turning angular velocity, and the mathematical relationship between the scraper speed and steering angular velocity;
(2) For the selection of control scheme, based on the kinematics model of the articulated LHD lateral error identifying the scraper and heading angle error, the error between the steering angle and the curvature of state space, according to the state space, is put forward to the steering angle control to control the amount of articulated LHD vehicle location of LQR controller, linear quadratic linear optimal control;
(3) Aiming at the problem of the difficult parameter selection of the LQR controller, we propose the LQR controller scheme optimized by the intelligent cluster algorithm, compare the advantages and disadvantages of different clustering algorithms, and put forward a feasible implementation scheme for path tracking control of the intelligent scraper.
(4) This paper serves as a guide to starting a conversation; we did not clearly indicate which algorithm (AGA, OPSO, or ACA) is better, because each of them has its own advantages and disadvantages. We will continue research in this direction and hope more and more researchers will be interested in this direction as well.

## Figures and Tables

**Table 1:** Articulated shovel vehicle body parameters.

Parameter Name	Numerical Value
Distance from front bridge to the articulation point ($L_{1}/m$)	1.766
Rear bridge distance to articulation point distance $\left(L_{2}/m\right)$	1.866
Tire diameter (d/m)	1.32
Body width (W/m)	2.27
Articulated steering angle change range (γ/rad)	0.30π
Maximum speed $V_{\max}$ (m/s)	7.2
Maximum steering angle speed change range ($\gamma_{\max}/\mathrm{rad}\times s^{-1}$)	0.17

**Table 2:** AGA parameter configuration.

Parameter Name	Numerical Value
Population size: N	30
Iterations: G	50
Cross-crossing probability	0.2
Adaptive variation constant	0.2
Variation constant b	3
Population survival range	0–50

**Table 3:** AGA algorithm searching for optimal results.

Algorithm	Weighted Matrix Q			Linear Feedback Matrix K			Suitability
	$q_{1}$	$q_{2}$	$q_{3}$	$k_{1}$	$k_{2}$	$k_{3}$	
AGA	1.4685	33.5161	33.8515	1.0605	6.3752	6.5008	18,220

**Table 4:** QPSO configuration of quantum behavior particle group algorithm parameters.

Parameter Name	Numerical Value
Iterations: G	80
Population size: N	30
Termination of the inertia weight $w_{\mathrm{end}}$	0.4
Initial inertia weight $w_{\mathrm{ini}}$	0.9
Learning factor $a_{1}$	1.5
Learning factor $a_{2}$	1.5
Particle taking value limit $q_{\max}$	400
Maximum speed $v_{\max}$	1.0
Initial shrinkage factor of expansion $\alpha_{b}$	1.0
Termination shrinkage—expansion factor $\alpha_{e}$	0.5

**Table 5:** Optimization results of the QPSO algorithm.

Algorithm	Weighted Matrix Q			Linear Feedback Matrix K			Suitability
	$q_{1}$	$q_{2}$	$q_{3}$	$k_{1}$	$k_{2}$	$k_{3}$	
QPSO	40.8844	49.0588	43.0995	5.2700	10.6900	6.7451	17,330

**Table 6:** ACA parameter configuration.

Parameter Name	Numerical Value
Ant number: ant	30
Search times: G	100
Hormone play factor $w_{ini}$	0.4
Transfer probability $P_{0}$	0.2

**Table 7:** ACA algorithm optimization results.

Algorithm	Weighted Matrix Q			Linear Feedback Matrix K			Suitability
	$q_{1}$	$q_{2}$	$q_{3}$	$k_{1}$	$k_{2}$	$k_{3}$	
ACA	0.8419	7.0752	40.6476	0.8288	4.1523	6.5550	15,333

**Table 8:** Parameter configuration comparison.

Algorithm Name	Population Size	Number of Convergence Iterations	Operation Time
Adaptive genetic algorithm (AGA)	30	50	15 min
Quantum behavior particle swarm algorithm (QPSO)	30	80	25 min
Ant colony algorithm (ACA)	30	No convergence	30 min

**Table 9:** Comparison of algorithm optimization results.

Algorithm	Weighted Matrix Q			Linear Feedback Matrix K			Suitability
	$q_{1}$	$q_{2}$	$q_{3}$	$k_{1}$	$k_{2}$	$k_{3}$	
AGA	1.4685	33.5161	33.8515	1.0605	6.3752	6.5008	18,220
QPSO	40.8844	49.0588	43.0995	5.2700	10.6900	6.7451	17,330
ACA	0.8419	7.0752	40.6476	0.8288	4.1523	6.5550	15,333

## Data Availability

Not applicable.
